# A Systematic Review on Chinese Baijiu Aging Process in Cellular Built Environment: States, Challenges, and Directions

**DOI:** 10.1002/fsn3.71742

**Published:** 2026-04-07

**Authors:** Jin Li, Yuwen Wen, Chang Yi, Wenwu Zhou, Yin Zhang

**Affiliations:** ^1^ School of Civil Engineering Sichuan University of Science & Engineering Zigong China; ^2^ School of Architecture and Design Southwest Minzu University Chengdu China; ^3^ College of Chemistry and Environment Engineering Sichuan University of Science & Engineering Zigong China

**Keywords:** “Gas‐Thermal‐Humidity”, aging process, cellar, Chinese Baijiu

## Abstract

The aging of Chinese Baijiu is a critical determinant of both product quality and production efficiency, yet the role of cellar microenvironments in shaping this process remains insufficiently understood. In particular, the interactions among thermal conditions, humidity, and volatile compounds released from Baijiu have been largely overlooked, leaving gaps in understanding the mechanisms that govern aging dynamics. This study synthesizes current research on Baijiu aging environments, encompassing environmental factors, regulatory strategies, and dynamic monitoring techniques, and introduces the concept of a “Gas‐Thermal‐Humidity” microenvironment. This framework emphasizes the coupled effects of volatile compounds and ambient conditions, offering a comprehensive perspective on microenvironment formation. Empirical observations reveal substantial spatial and temporal variability in underground cellar temperatures, while the reciprocal impact of volatiles on the surrounding environment remains insufficiently studied. To address these gaps, a multi‐faceted research framework is proposed, including dynamic measurement of key cellar parameters, statistical analysis to identify spatiotemporal patterns, development of a coupled gas‐thermal‐humidity multi‐physical field model with numerical solutions, and construction of a multi‐scale, multi‐parameter evaluation method for cellar microenvironments. By integrating engineering thermophysical approaches such as computational fluid dynamics, thermal and humidity analysis, and multi‐field coupling, this study provides novel insights into the mechanisms of Baijiu aging, along with practical strategies for optimizing production, thereby contributing both theoretical advances and applied value to the field.

## Graphical abstract

This review addresses a critical gap in Chinese Baijiu aging research: the neglect of physical properties in cellar storage environments. Current studies emphasize microorganisms, CO_2_, and base liquor interactions, yet lack systematic consideration of environmental dynamics. To bridge this gap, the “Gas‐Thermal‐Humidity” coupled microenvironment is proposed as a core concept. An integrated framework is further established, combining multi‐parameter monitoring (temperature, humidity, airflow, CO_2_, aroma compounds) with statistical analysis, multi‐physics modeling of heat and mass transfer, and AHP‐FCE‐based comprehensive evaluation. This framework offers a science‐driven pathway to optimize aging conditions.

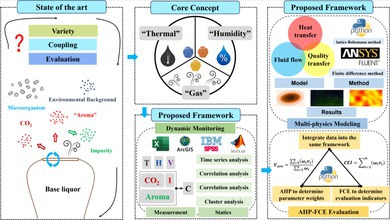

## 1 Introduction

As one of the world's three major distilled spirits, Chinese Baijiu possesses a long history (Fan and Xu 2023) and is renowned internationally for its diverse and distinctive aroma profiles, including Moutai, Luzhou, and Light flavors (Xiao et al. 2016). Traditionally, Baijiu is produced from raw materials such as sorghum, wheat, and corn, with *qu* serving as both saccharification and fermentation starter. Its production involves four key stages: fermentation (Liu and Sun 2018), distillation (Li et al. 2012), aging (Rizeng et al. 2023), and blending (Wang 2024). Among these, aging is particularly critical, as freshly distilled base Baijiu exhibits a sharp, pungent taste due to high concentrations of fermentation by‐products. Prolonged storage mitigates these harsh flavors, resulting in a smoother mouthfeel and enhanced aromatic complexity (Wang et al. 2024). However, aging is inherently time‐consuming, imposing substantial economic burdens on producers and complicating quality and safety management (He et al. 2023). Consequently, accelerating the aging process has become a central research focus, offering potential solutions to both economic and quality challenges in Baijiu production.

### 1.1 Aging Theory

Understanding the aging mechanism is essential for developing precise strategies for Chinese Baijiu maturation. Researchers have examined the process from multiple perspectives, and several comprehensive reviews have summarized the evolution of mechanistic hypotheses (He et al. 2023). The main theories include the Association (Peng 2021), Esterification (Sun et al. 2022), Leaching (Qiao 2014), Volatilization (Zhu and Cadwallader 2019), and Oxidation (Deng et al. 2020) theories. The Association Theory proposes that ethanol‐water interactions reduce free ethanol availability, thereby elevating its boiling point. The Esterification Theory emphasizes that ester formation between aromatic compounds and ethanol contributes substantially to aging (Niu et al. 2017). According to the “Leaching Theory”, trace metal ions from the container play a crucial role in the maturation process (Huang et al. 2020). According to the Volatilization Theory, the gradual release of sulfides diminishes pungent flavors (Zhu et al. 2020), while the Oxidation Theory attributes aging to the slow oxidation of alcohols (Gao et al. 2014). Although these hypotheses remain incompletely validated and have limitations, they encapsulate extensive empirical observations and constitute a valuable knowledge base with significant practical guidance.

### 1.2 Aging Techniques

#### 1.2.1 Natural Aging

Natural aging refers to the maturation of Baijiu without artificial physical, chemical, or biological interventions, relying instead on appropriate storage conditions and temperature control. Common approaches include outdoor (Gao 2016), warehouse (Sun et al. 2022), cellar (Zheng et al. 2016), and cave (Yang et al. 2011) aging. Although time‐consuming, natural aging typically yields superior quality compared to artificial methods. To date, no artificial technique has fully replicated the effects of natural aging (Qiao 2013), highlighting the importance of developing strategies to accelerate this process.

#### 1.2.2 Artificial Aging

The mechanisms underlying Baijiu aging remain largely speculative due to limited empirical evidence and a lack of rigorous methodologies. Existing hypotheses primarily focus on the internal characteristics of Baijiu, often neglecting the influence of external thermal and humidity conditions. Natural aging involves not only complex internal physical and chemical reactions and microbial activities but also the properties of ceramic containers, the physicochemical attributes of Baijiu, and interactions between microorganisms and the storage environment. As a result, elucidating the precise mechanisms of Baijiu aging remains a considerable challenge.

Concurrently, significant progress has been made in developing aging methods, broadly classified as physical, chemical, and biological techniques (Zhang et al. 2023). However, most of these approaches are temporary, have not seen widespread industrial adoption (Fan 2023), and fail to fully replicate natural aging (Qiao 2013). While Baijiu is not produced in Western countries, their extensive experience with wine aging highlights the inadequacy of isolating storage conditions in aging studies; some scholars have proposed the concept of “wine‐barrel‐cellar” interactions (Roussey et al. 2020). This underscores that research in China lags behind, largely due to insufficient engineering focus on controlling temperature and humidity. With recent advancements in indoor environment monitoring technologies, investigating Baijiu aging from the perspective of storage space conditions has become increasingly imperative.

### 1.3 Limitations of Existing Research

Although there has been extensive exploration of certain aging theories and technologies, there are still some fundamental limitations in the existing research on the aging of Chinese Baijiu. Current mechanistic hypotheses and practices mainly regard Baijiu aging as a process of chemical reactions and physical transformations within the Baijiu. Therefore, research has primarily focused on the internal conditions of Baijiu, while considering the storage environment as a secondary factor.

However, the aging of Baijiu is essentially a long‐term process that occurs through continuous interaction with the surrounding physical environment. Factors such as ambient temperature, humidity, container permeability, and the coupled thermal and moisture conditions of the storage space directly affect mass transfer, reaction kinetics, and microbial activity. Ignoring these external conditions not only limits the explanatory power of aging theories for Baijiu but also increases the difficulty of artificially reproducing or accelerating natural aging. Therefore, current research tends to overemphasize the internal conditions of the Baijiu while neglecting the external physical conditions, which restricts both theoretical validation and practical applications. To address this imbalance, it is necessary to systematically reassess Baijiu aging from the perspective of the external physical environment of the storage space.

### 1.4 Research Gap and Focus

Despite the development of various techniques to accelerate Baijiu aging, existing studies have predominantly focused on the internal conditions of the base Baijiu, failing to provide a comprehensive evaluation of the external thermal and humidity environment. Meanwhile, these approaches overlook the critical interplay among the physical properties of ceramic vessels, the physicochemical characteristics of the Baijiu, and the surrounding cellar environment. This oversight neglects the scientific reality that natural Baijiu aging depends not only on intricate physicochemical reactions and microbial metabolic processes within the Baijiu but also on the coupled interactions with external environmental factors. Critically, the direct, mechanistic evidence for these couplings—particularly how volatilized organics influence the cellar's thermal‐humidity environment and vice‐versa—remains largely unquantified.

To address this significant gap, the present study highlights the significance of the aging environment's physical properties and proposes innovative, technology‐driven methods for accelerating aging. First, existing studies on environmental factors, control mechanisms, optimal condition creation, and dynamic monitoring techniques are systematically reviewed. Building on this foundation, a cognitive framework is established, and key challenges and bottlenecks in current research are identified. Finally, the concept of a “Gas‐Thermal‐Humidity” microenvironments is introduced, providing a detailed research framework and practical implementation strategies to guide future innovations.

## 2 Current Status

Chinese Baijiu is usually aged in large ceramic containers for a long time under natural storage conditions, which makes its aging system different from that of wine. The aging environment of Baijiu is characterized by the complex interaction among storage containers, environmental conditions, and physical and chemical transformation processes.

One of the most remarkable characteristics of Baijiu aging is the use of traditional ceramic storage containers. Ceramic containers have a microporous structure that allows for limited gas exchange between the internal liquid phase and the external environment. This structural characteristic slows down oxygen diffusion and promotes the adsorption desorption process of volatile compounds on the container surface, which can regulate the concentration of aromatic active compounds and facilitate physical and chemical transformation during long‐term storage. Moreover, the permeability and surface properties of ceramic materials can affect the evaporation process, leading to changes in alcohol content and volatile components over time.

Another important aspect of Baijiu aging is the gradual transformation of volatile components. During long‐term storage, alcohols, esters, aldehydes, and organic acids in alcohol undergo a series of chemical reactions, including esterification, oxidation, and hydrolysis. These reactions lead to a gradual adjustment of aroma components, typically resulting in smoother and more mellow flavors. The research shows that aging can reduce the irritation of fresh Baijiu and increase the complexity of aroma compounds. Although the alcohol concentration in Baijiu will restrict the growth of microorganisms in the liquid phase, microorganisms may still exist in the surrounding storage environment or container surface. These microbial communities can indirectly affect the aging process by interacting with environmental conditions to produce trace metabolites. However, the research on microbial ecology in Baijiu aging environment is still relatively limited, and further research is needed.

In a word, the aging environment of Baijiu is a complex system, involving the interaction between container materials, environmental conditions, and chemical conversion processes. Understanding these interactions is crucial to reveal the aging mechanism of Baijiu and provides an important basis for developing a system framework to describe the gas heat moisture coupling effect in the aging system.

Although a direct correlation between the storage environment and ripening quality has not been definitively established, the academic community has increasingly recognized the significance of the cellar storage environment in the aging process of base wine and has conducted extensive research on this topic (Arredondo‐Ruiz et al. 2020). Given the limited focus on cellar storage environments specific to our country, many perspectives are drawn from wine aging studies. The research in this area primarily encompasses five key aspects:

### 2.1 Environmental Factors

#### 2.1.1 Temperature

Research on temperature can be summarized in two key aspects. Firstly, it involves monitoring variations in environmental temperature when utilizing spaces like basements and caves as aging rooms. Secondly, it focuses on maintaining a stable environment to observe variations in color (Hopfer et al. 2013), flavor (Jung et al. 2021), compositions (Cutzach et al. 2000; Arapitsas et al. 2014; Drappier et al. 2019; Scrimgeour et al. 2015), wine losses (Ruiz de Adana et al. 2005), gases (Cejudo‐Bastante et al. 2013), and microbial communities of stored items (Kim et al. 2023; Belda et al. 2017), especially beverages. However, the need for stable temperatures in storage rooms often results in higher energy consumption (Benni 2014). Table 2 presents the measured temperature values from various natural wine cellars, highlighting significant variations in temperature fluctuations. These differences are primarily driven by the combined effects of cellar structure, soil properties, and outdoor meteorological conditions.

Moreover, (Cañas Guerrero and Martin Ocaña 2005) studied the hygro‐thermal behavior of two traditional underground wine cellars in Morcuera (Soria, Spain). (Cejudo‐Bastante et al. 2013) found that temperature factors (storage and accelerated aging) induced common changes in the volatile profiles of Chardonnay wines. (Martín Ocaña and Cañas Guerrero 2006) highlighted the uneven temperature distribution in wine cellars, while (Barbaresi, de Maria, et al. 2015) observed that indoor temperatures in aboveground buildings are more uniform. (Silvia and Ignacio 2005) examined the impact of temperature and humidity differences within wine cellars on wine quality. (Igarashi et al. 2019) demonstrated that adjusting temperature and humidity in an artificial climate room could alter wine taste evaluations. (Hopfer et al. 2013) studied the combined effects of storage temperature and packaging on Californian Cabernet Sauvignon, finding that elevated temperatures could be useful for wine packaging screening. (Mazarrón and Cañas 2023) monitored 19 Spanish wine cellars, revealing significant variability in hygrothermal conditions. Additionally, (Ocón et al. 2011) identified temperature and humidity as key factors influencing the presence of mold in the air. Consequently, studying the cellar and wine as an integrated system is essential for a more comprehensive understanding of their interactions and dynamics.

Table 1 summarizes the reported temperature ranges for different Baijiu storerooms and warehouses. Although underground storage environments are generally considered relatively stable, collected data indicates that significant temperature fluctuations still occur due to various factors, including seasonal climate change, ventilation conditions, structural characteristics of storage facilities, and thermal inertia of surrounding soil. These factors result in differences in the magnitude and pattern of temperature changes between different storage systems. Temperature fluctuation will affect the physical and chemical process of Baijiu aging. For example, temperature can affect the rates of chemical reactions such as esterification, hydrolysis, and oxidation, as well as the volatility and diffusion of aromatic compounds. Even moderate changes in temperature may alter the balance of mass transfer processes and volatile components in storage environments. Therefore, the temperature characteristics summarized in Table 1 are not only descriptive data, but also provide an important empirical basis for understanding the environmental dynamics of Baijiu aging. These observations further advance the development of the gas heat moisture coupling framework proposed in this study, which aims to systematically describe the interactions between temperature, humidity, and gas transport processes in aging environments.

**TABLE 1:** Indoor air temperatures of different aging rooms.

Wine cellars or resources	Indoor air temperature (°C)		
	Average	Minimum	Maximum
Basement cellar^a^	16.4	11.3	26
Morceura (Top and Summer)^b^	11.8	11.4	12.4
Morceura (Bottom and Summer)^b^	9.0	9.0	9.0
Morceura (Top and Winter)^b^	7.1	7	7.4
Morceura (Bottom and Winter)^b^	6.6	6.2	6.8
Alcubilla del Marques (Eugenia and Summer)^b^	12.1	11.8	12.6
Alcubilla del Marques (Teofilo and Summer)^b^	11.5	11.4	12.2
Alcubilla del Marques (Eugenia and Winter)^b^	12.5	12.2	12.9
Alcubilla del Marques (Teofilo and Winter)^b^	10.1	9	11
Underground cellar^c^	9.9	8.6	11.1
Basement cellar^c^	13.2	8.9	17.3
Aboveground without Air‐conditioning^c^	14.4	7.8	21.8
Earth‐sheltered^c^	12.5	10.3	14.9

^a^ Data from Barbaresi et al. (2014).

^b^ Data from Cañas Guerrero and Martin Ocaña (2005).

^c^ Data from Geyrhofer (2011).

#### 2.1.2 Humidity

Scholars commonly study temperature and humidity together, as monitoring instruments typically measure both parameters simultaneously (Morais et al. 2018). Unlike temperature, relative humidity in underground cellars exhibits less fluctuation and tends to be more uniform across different locations. For example, a study by (Cañas et al. 2022) on cider aging found that relative humidity generally ranged from 60% to 90%. (Gómez‐Villarino et al. 2021) reported average humidity levels of 77%, 80%, and 94% for basement, buried, and underground cellars, respectively. Additionally, (Mwithiga et al. 2013) developed a humidity control system to maintain optimal humidity levels in cellars. Theoretically, relative humidity influences the volatilization rate of the wine, and conversely, wine evaporation affects the indoor humidity levels. The coupling relationship between these two factors underscores the importance of further investigation into effective cellar humidity control.

#### 2.1.3 Gas

Gas transfer, and especially oxygen, through wine closures has been studied and confirmed since the 1990s (Crouvisier‐Urion et al. 2018). The gases in the cellar environment also originate from the volatilization of compounds within the wine itself. Accordingly, this study reviews research on the volatile gases present in the wine, as summarized in Table 2. Most existing studies focus on the concentration changes of various volatile gases, such as oxygen, carbon dioxide, and aroma compounds, in base wine of different ages, using these changes to infer the effects of external temperature and humidity control measures. However, conclusions can vary depending on the type of wine being studied. While research on Baijiu remains limited, most studies focus primarily on the changes in aroma components within the Baijiu. Furthermore, the majority of studies concentrate on the gases inside the Baijiu itself, with few investigating how the volatilization of these gases into the surrounding air impacts the thermal and humidity dynamics of the cellar environment.

**TABLE 2:** Gases studied in different literature.

Resources	O_2_	CO_2_	SO_2_	H_2_S	MeSH	DMS	VOCs	NO_2_
Wang et al. (2022)	■							
Panaras et al. (2021)		■					■	■
Toussaint et al. (2014)	■	■						■
Cutzach et al. (2000)							■	
Ugliano et al. (2012)				■	■	■		
Ferreira et al. (2014)			■	■	■	■		
Tarasov et al. (2021)	■	■	■					
Ferreira et al. (2023)				∎	■			
Vidal and Moutounet (2006)	■							
Franco‐Luesma and Ferreira (2016)				■	■	■		
Dimkou et al. (2011)	■		■					
Flor et al. (2018)		■						

*Note:* ∎ indicates that the gas parameter was investigated in the corresponding study. Blank cells indicate that the parameter was not investigated.

Abbreviations: DMS, Dimethylsulfide; MeSH, Methanethiol.

Moreover, (Geyrhofer 2011) investigated airflow velocity in underground wine cellars and determined that existing air outlets often fail to generate adequate convection, emphasizing the need to minimize improper ventilation practices. Barbaresi et al. employed the lattice Boltzmann method to numerically simulate airflow dynamics during the wine aging process, analyzing how room layout and air vent configurations affect overall air circulation (Barbaresi 2015). (Barbaresi et al. 2014) conducted continuous temperature measurements in an underground wine cellar in Italy over the course of a year, revealing a temperature variation of up to 14.7°C within the cellar. This underscores that not all underground wine cellars can maintain a consistent temperature. In contrast, Silvia et al.'s monitoring of traditional underground wine cellars in the Tierras Sorianas del Cid region of Spain demonstrated that cellar temperatures were generally more stable compared to external temperatures (Silvia and Ignacio 2005). Guerrero and Ocaña explored the thermal and humidity behavior of two traditional underground wine cellars in the Morqueira region of Soria Province, Spain, and speculated on the effects of temperature differentials on the aging process (Cañas Guerrero and Martin Ocaña 2005). These studies represent a shift from empirical, “black box” approaches to a more scientific, “white box” perspective on optimizing cellar environments, marking a significant advancement in the scientific understanding of aging. However, focusing solely on improvements in ventilation, temperature, and airflow fields does not fully address the complex interplay between multiple physical factors, leading to recommendations that may lack comprehensiveness.

### 2.2 Environmental Regulation

Ventilation control is a critical aspect of managing the cellar environment. Santolini et al. (2019) utilized Computational Fluid Dynamics (CFD) simulations to optimize ventilation strategies for underground wine cellars. Barbaresi, Torreggiani, et al. (2015) identified temperature inhomogeneity in these cellars and developed a temperature prediction method to assess the degree of temperature field uniformity, thereby enhancing monitoring schemes. In response to temperature field inconsistencies, they also designed a micro‐ventilation system aimed at improving temperature and humidity uniformity without altering the average temperature (Barbaresi et al. 2020). Additionally, Mazarrón et al. examined the impact of traditional ventilation openings, known as zarceras, on the thermal and humidity properties of underground wine cellars in Spain, demonstrating that such systems can support optimal conditions for wine maturation without the need for air conditioning (Cañas and Mazarrón 2009). They also noted that the thermal inertia of the surrounding ground can create favorable temperature conditions (Mazarrón et al. 2012b). (Jiménez‐López et al. 2024) proposed a bioclimatic strategy to decrease energy consumption by using a quantitative theoretical method. They recommended integrating high thermal mass and shading techniques. Despite these valuable contributions, the research predominantly treats underground wine cellars as typical indoor environments. It often overlooks the interactive nature of the internal environment and the barrels themselves. Future studies should integrate these components to provide a more holistic understanding of cellar dynamics and improve environmental control strategies.

### 2.3 Formation Mechanism

Indoor temperatures in underground wine cellars typically exhibit less fluctuation and greater stability compared to above‐ground structures. It is largely attributed to the thermal inertia of the surrounding soil, which moderates temperature variations and contributes to energy savings (Mazarrón et al. 2012b, 2012a; Tinti et al. 2015, 2014; Rodríguez‐Gonzálvez et al. 2014; Martin and Canas 2006; Tinti 2017). Martín Ocaña and Cañas Guerrero (2006) investigated the thermal properties of traditional underground wine cellars in Spain and proposed a straightforward model for predicting cellar temperatures. While this model can estimate the indoor temperature range, it is inadequate for predicting temperature variations over time. The significant thermal inertia of the soil allows for boundary conditions to be based primarily on seasonal temperature changes, rendering outdoor meteorological factors minimal in their influence.

Furthermore, some studies have entirely bypassed physical investigations in favor of developing predictive models. Mazarrón and Cañas (2008), for example, formulated a mathematical model specifically for traditional underground wine cellars in Spain's “Ribeira de Duero” region. Despite these advancements, there remains a notable gap in research regarding the formation mechanisms of thermal and humidity environments in underground cellars. Specifically, there is a lack of studies exploring the multiphysics coupling effects involving internal disturbances and the volatilization dynamics of the building envelopes, indicating a need for more scientifically rigorous investigations.

Additionally, this study summarizes the calculations for soil temperature, as presented in Table 3. Soil temperature is a critical boundary condition and a key factor influencing the accuracy of simulations of the thermal environment in wine cellars. Currently, most researchers use periodic soil boundary conditions, which approximate soil temperature fluctuations through Fourier series in complex functions. Some modifications have been made to adapt these models to different regions. However, research specific to the Chinese context remains relatively scarce, highlighting the need for further investigation in this area.

**TABLE 3:** Calculation formula of soil temperature in the existed literature.

Formula	Resources
$T\left(x,t\right)=\left(T_{m}\right)-A_{s}e^{-x\sqrt{\frac{\pi }{365\alpha }}}\cos\left[\frac{2\pi }{365}\left(t-t_{0}-\frac{x}{2}\sqrt{\frac{365}{\pi \alpha }}\right)\right]$	Labs (1982)
$T\left(x,t\right)=T_{m}-A_{s}e^{-x\sqrt{\frac{\pi }{365\alpha }}}\cos\left\{\frac{2\pi }{365}\left[\left(t-t_{0}-\frac{x}{2}\right)\sqrt{\frac{365}{\pi \alpha }}\right]\right\}$	Kusuda and Achenbach (1965)
$T\left(x,t\right)=\left(T_{m}-k\right)-A_{s}e^{-x^{\prime }\sqrt{\frac{\pi }{365\alpha }}}\cos\left[\frac{2\pi }{365}\left(t-t_{0}^{\prime }-\frac{x^{\prime }}{2}\sqrt{\frac{365}{\pi \alpha }}\right)\right]$	Mazarrón and Cañas (2008)
$T\left(x,t\right)=9.76-9.65e^{-0.31x}\cos\left[\frac{2\pi }{365}\left(t-35-18.06x\right)\right]$	Al‐Temeemi and Harris (2001)
Tx,t=1hehrTma−ϵΔR+bSm−0.0168hsfb1−ra+hrAsa−bSaexpiφ1−φa/he+KsReexpiwt	Mihalakakou et al. (1997)
$T\left(x,t\right)=T_{m}+T_{c}\left(-A_{s}+A_{c}\right)e^{-x\sqrt{\frac{\pi }{365\alpha }}}\cos\left\{\frac{2\pi }{365}\left[\left(t-\left(t_{0}+t_{c}\right)-\frac{x}{2}\right)\sqrt{\frac{365}{\pi \alpha }}\right]\right\}$	Tinti et al. (2014)
$T\left(x,y,z,t\right)=T_{\mathrm{me}}\left(x,y\right)-1.07S_{h}A_{e}\left(x,y\right)\exp\left[-z\sqrt{\frac{\pi }{365\alpha \left(x,y,z\right)}}\right]\cos\left\{\frac{2\pi }{365}\left[t-t_{T_{0}}\left(x,y\right)-\frac{z}{2}\sqrt{\frac{365}{\pi \alpha \left(x,y,z\right)}}\right]\right\}+\frac{h\left(x,y\right)}{\lambda \left(x,y,z\right)}z$	Baggs (1983)
$T_{g}\left(x,y,z,t\right)=T_{m}\left(x,y\right)+k\left(x,y\right)-\eta \left(x,y\right)A_{e}\left(x,y\right)\exp\left[-z\sqrt{\frac{\pi }{365\alpha \left(x,y,z\right)}}\right]\cos\left\{\frac{2\pi }{365}\left[t-t_{T_{0}}\left(x,y\right)-\tau \left(x,y\right)-\frac{z}{2}\sqrt{\frac{365}{\pi \alpha \left(x,y,z\right)}}\right]\right\}+\frac{h\left(x,y\right)}{\lambda \left(x,y,z\right)}z$	Tinti (2017)
${\left.\bar{T}\left(x,y\right)=\left[T_{m}\left(x,y\right)+k\left(x,y\right)\right]+\frac{\mathrm{A\pi}}{\left[s\left(x,y\right)-r\left(x,y\right)\right]\cdot 2\cdot \sqrt{\frac{\pi }{365\alpha \left(x,y,z\right)}}}\exp\left[-z\sqrt{\frac{\pi }{365\alpha \left(x,y,z\right)}}\right]\left\langle \left\{\sin\frac{2\pi }{365}\left[t-t_{T_{0}}^{,}\left(x,y,z\right)-\frac{z}{2}\sqrt{\frac{365}{\pi \alpha \left(x,y,z\right)}}\right]\right\}+\cos\left\{\frac{2\pi }{365}\left[t-t_{T_{0}}^{,}\left(x,y,z\right)-\frac{z}{2}\sqrt{\frac{365}{\pi \alpha \left(x,y,z\right)}}\right]\right\}\right\rangle \right|}_{r}^{s}$	Tinti (2017)

### 2.4 Recommendations

The relationship between temperature, humidity, and aging has been a subject of interest in brewing for centuries. Since the 1950s, research in various countries has investigated the optimal environmental conditions for winemaking. Current recommendations suggest maintaining a temperature range of 9°C–20°C (Troost 1953), a thermal variation of less than 6°C (Vogt 1971), and a relative humidity exceeding 70% (Troost 1953). Additionally, it is crucial to consider the impact of temperature and humidity on the evaporation loss of base wine (Nègre et al. 1965). While, Scrimgeour et al. (2015) pointed out that there is no “ideal” storage temperature for wine in general. As for Baijiu, Niu et al. (2022) recommended a temperature range of 9°C–20°C and a relative humidity exceeding 70%. It is evident that many studies have relied on recommended values for temperature and humidity; however, these recommendations have often not been rigorously validated. This study contends that the exploration of optimal temperature and humidity conditions for aging different types of Baijiu should extend beyond current assumptions. Further investigation is essential to substantiate and refine these parameters, ensuring more reliable and accurate guidelines.

In China, optimal temperature and humidity ranges for aging have also been proposed, largely based on engineering experience rather than empirical research. Although early investigations laid the groundwork, progress has been limited due to entrenched beliefs in established guidelines within the brewing industry. This adherence to conventional recommendations has hindered further exploration into the mechanisms by which temperature, humidity, and airflow affect the aging process. Based on these observations, we recommend that future research should go beyond empirical approaches and strive to establish quantitative correlation models between “environmental parameters‐physical field characteristics‐aging quality indicators,” Such models would enable a more rigorous and predictive understanding of how temperature, humidity, and airflow collectively influence the maturation of Baijiu, providing a more scientific basis for optimizing cellar conditions.

### 2.5 Dynamic Monitoring

The primary objective of monitoring is to promptly identify and rectify anomalies, ensuring both accuracy and correctness. Effective monitoring necessitates precision, with contemporary approaches heavily relying on automation technology. Despite the relatively recent initiation of Baijiu storage environment monitoring in China (Kurniawan and Witanti 2021), the rapid advancement of technologies such as the Internet of Things (IoT) has facilitated the widespread adoption of automated monitoring systems in production enterprises. Current research has concentrated on enhancing monitoring accuracy through improved sensor precision and algorithm optimization, employing various technologies (Table 4).

**TABLE 4:** Monitoring technologies in Baijiu production and storage.

Technologies	Type	Parameters	Resources
NB‐IoT + Zigbee+Ali Cloud	Chinese Baijiu	T, CC	Luo and Shi (2023b)
fuzzy PID + Zigbee	Chinese Baijiu	T, CC	Luo and Shi (2023a)
PLC + LabView	Wine	T, H, I	Zeng (2020)
AI+fuzzy PID	Wine	T, H, I	Zeng (2021)
NB‐IoT	Wine	T, H, I, EC, IAT	Li and Wang (2020)
ACOA	Chinese Baijiu	T, H, PH, M	Shi (2017)
RFID	Chinese Baijiu	T, H	Fu (2019)
ZigBee	Chinese Baijiu	T, H	Dong (2020)
CAN‐bus	Chinese Baijiu	T, H	Wang (2009)

Abbreviations: ACOA, advanced ant colony optimization algorithms; CC, CO_2_ concentration; EC, Ethanol concentration; H, Humidity; I, Illuminance; IAT, Indoor air quality; M, microorganism; T, Temperature.

Research on monitoring parameters has predominantly focused on temperature and humidity. While these studies have made significant contributions to enhancing monitoring accuracy and reducing measurement noise, challenges remain in achieving precise measurements. These challenges often stem from design assumptions that overlook the physical properties of the environment, leading to inaccuracies in initial diagnoses. Key issues include: Design assumptions typically presume a uniform indoor temperature field, neglecting field non‐uniformities introduced by the coupling of multiple physical fields. Continuous dynamic requirements are often not integrated with these non‐uniformities. Monitoring Considerations: There is frequently an inadequate consideration of the relationship between the storage environment and the aging process of Baijiu. The assumption that monitoring only temperature and humidity provides comprehensive oversight is often insufficient. Given the complexity of indoor environments, effective monitoring of storage conditions requires a foundation of scientific evaluation. Historical reviews of air environment evaluation indicate that current assessment systems have not extended to address continuous dynamic requirements in storage environments. Therefore, existing monitoring parameters may fail to capture critical aspects, and environmental control remains challenging. Further research is needed to optimize monitoring parameters and measurement point configurations under continuous dynamic conditions.

It should also be pointed out that the strong attention paid to temperature and humidity in the existing research largely reflects the research status of Baijiu aging environment. Most published studies mainly monitor these two parameters, while systematic datasets on other environmental variables, such as gas‐phase concentration gradients, volatile transport processes, or multi‐physics interactions, are still scarce. The existing literature provides limited empirical evidence for comprehensive synthesis beyond these common parameters. This situation highlights an important gap in the current research field. Therefore, expanding the analysis perspective from the traditional temperature and humidity monitoring to a more comprehensive description of gas transportation, thermal processes and humidity dynamics is a necessary step to promote the understanding of Baijiu aging environment.

## 3 Challenges

### 3.1 Environmental Specificity

The storage conditions for Chinese Baijiu are defined by their unique air dynamics, typically involving ceramic jars (Wang 2025) that do not fully isolate the Baijiu from the external environment, allowing for component exchange between the Baijiu and its surroundings. Key factors identified in current research include: the diffusion of aromatic compounds such as alcohols and esters into the air, creating an “atmosphere” that aids aging and needs to be maintained at optimal concentrations; the volatilization of impurities like sulfides, which contribute to undesirable “pungent” flavors and should be allowed to dissipate; the production of gases like CO_2_ from fermentation and microbial activity, which necessitates proper ventilation to prevent asphyxiation risks; and the role of mud seals on jars, which facilitate the transfer of microorganisms such as yeast and lactic acid bacteria between the Baijiu, mud, and air, influenced by temperature and humidity. This intricate microenvironment involves the interplay of volatile gases, ambient gases, and microorganisms (Gao et al. 2020), all contributing to the aging process of the Baijiu. Acquiring detailed data on the distribution of these gases in aging environments is essential for advancing research, yet significant gaps remain in our understanding of their distribution. This study also outlines the internal and external factors influencing the environment of different types of wine cellars. In underground cellars, periodic fluctuations like solar radiation and outdoor air have minimal impact on the environment.

Wine storage oak barrels are relatively airtight, largely isolating the liquid from the external environment, whereas Chinese Baijiu ceramic jars have a semi‐permeable structure. This semi‐permeable structure allows gas exchange between the Baijiu and the surrounding air, creating a dynamic microenvironment that affects the retention of volatile compounds, the transfer of microorganisms, and the aging process. These differences require a dedicated research framework for Baijiu aging, as barrels cannot replicate the unique “Gas‐Thermal‐Humidity” interactions present in ceramic jar systems.

**FIGURE 1:**
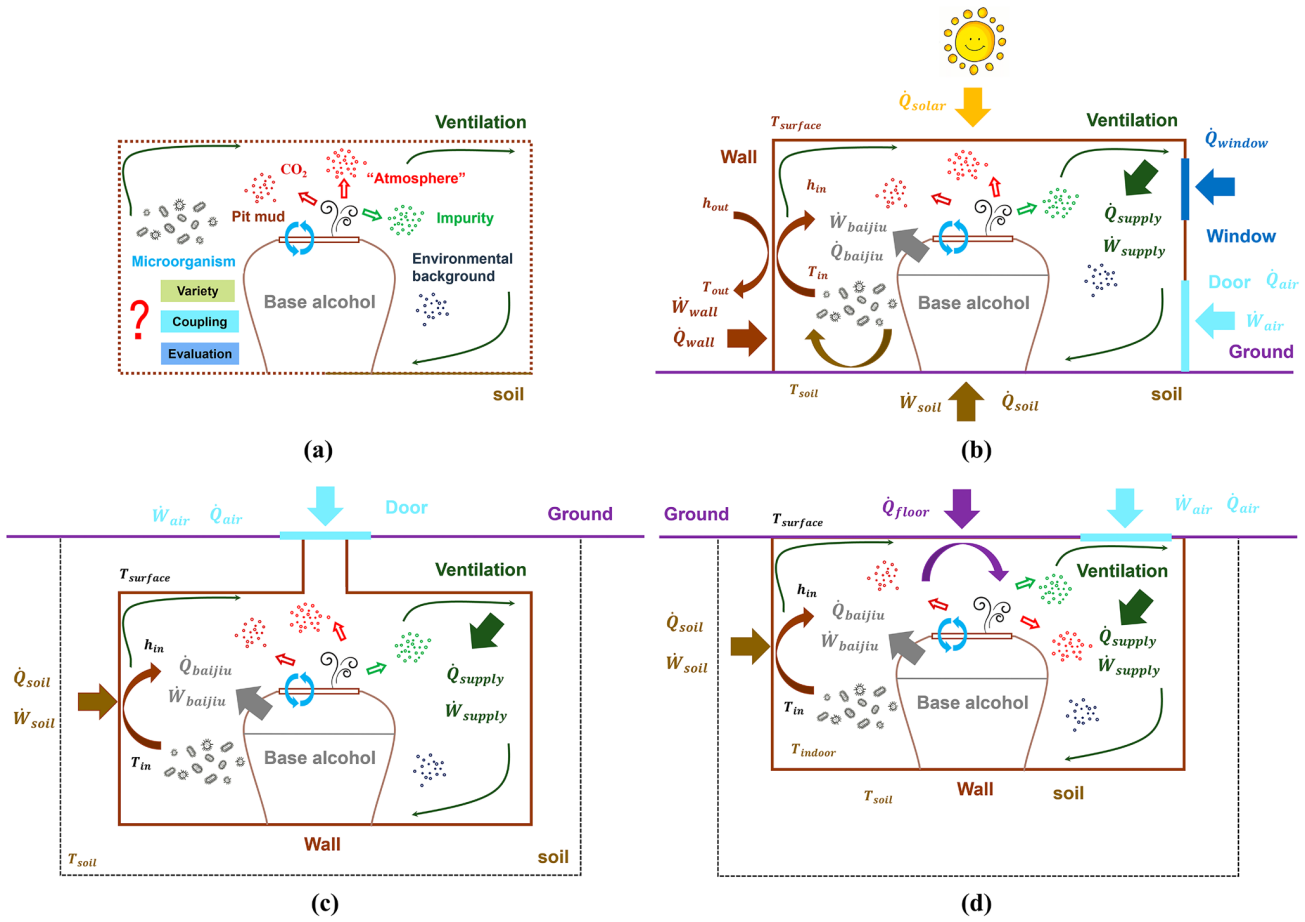
Diagram of the aging environment formation mechanism of Baijiu under different storage conditions: (a) Aging environment: Describing the direct relationship between the base spirit, atmosphere, microorganisms, and ventilation; (b) Aboveground: Solar radiation and outdoor air exchange are the main sources of thermal and disturbances; (c) Underground: The surrounding soil has minimal external disturbance; (d) Basement: A state influenced jointly by ground and indoor air disturbances. The four diagrams highlight the differences in thermal sources, disturbance mechanisms, and main environmental influencing factors under different typical aging scenarios.

Moreover, the “Gas‐Thermal‐Humidity” microenvironment may indirectly regulate the aging process by affecting the activity and community structure of microorganisms in the cellar soil and surrounding air. Changes in temperature, humidity, and gas concentrations can all modulate microbial metabolism and interactions, thereby influencing the transformation of aromatic compounds and the overall maturation of the Baijiu (Figure 1). This emphasizes that the environment is an interconnected component of the microbial system, highlighting the necessity of establishing a framework to study the aging of Baijiu.

#### 3.1.1 Mechanistic Interpretation of Gas‐Thermal‐Humidity Coupling

In this study, the proposed gas‐thermal‐humidity coupling concept describes the synergistic interaction among gas components, temperature, and humidity in a confined aging environment. “Gas” not only refers to the volatile compounds produced during the aging process but also to their dual role as carriers and reactants within the storage system. Furthermore, the concentration gradient of gaseous compounds in the container can serve as the fundamental driving force for mass transfer, facilitating the transmission and redistribution of volatile substances and oxygen, which subsequently participate in microbial metabolism and chemical reactions. Simultaneously, the transmission and reaction kinetics of these gases are directly influenced by the environmental temperature and humidity conditions, which jointly regulate the physical and chemical environment within the ceramic container. Their coupled effects affect the microporosity and permeability of ceramic materials, thereby influencing the gas diffusion pathways and the adsorption–desorption equilibrium on the container surface. Thermal‐humidity conditions control the rate of liquid evaporation and moisture migration, further altering the internal gas composition and reaction kinetics. Through these interrelated processes, gas transmission, microbial activity, and the formation of flavor compounds become dynamically coupled, forming a multi‐physical interaction mechanism: the gas field provides the material basis and reaction substrates, while the coupled thermal‐humidity field provides activation energy and physical pathways, jointly shaping the unique aging trajectory of Chinese Baijiu.

### 3.2 Challenges and Obstacles

**FIGURE 2:**
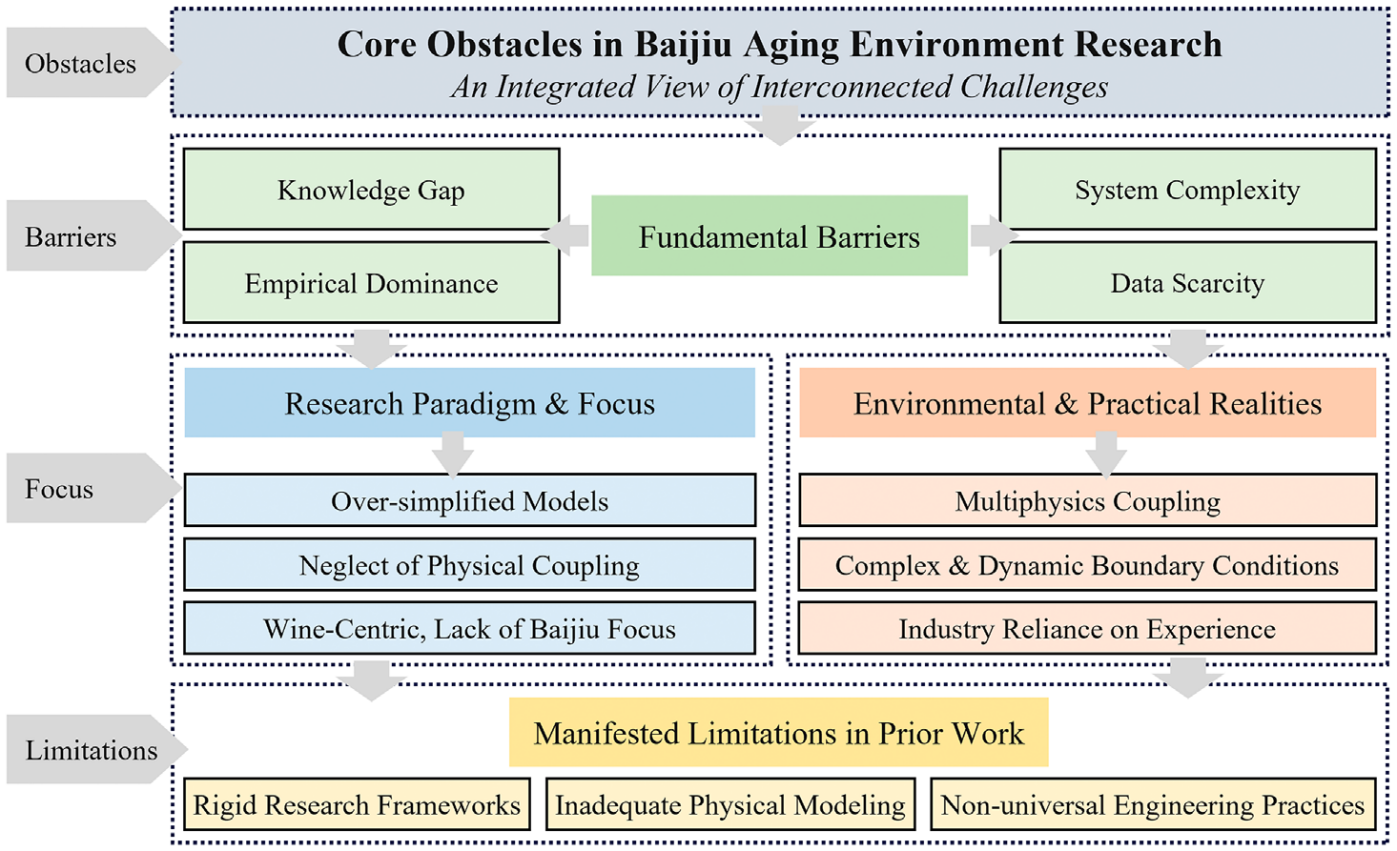
Summary of challenges and obstacles.

**FIGURE 3:**
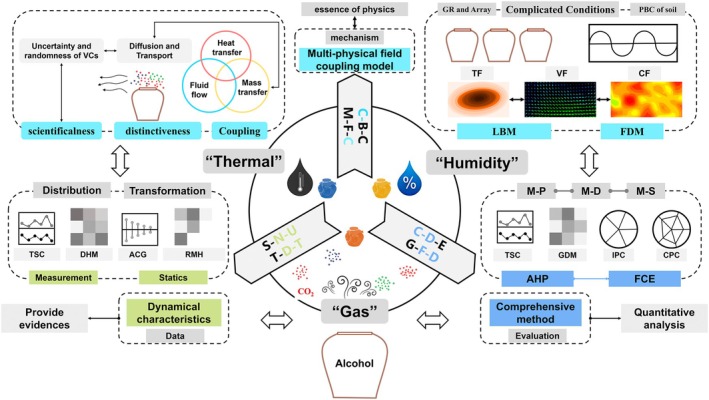
The novel framework to reveal the aging room environment. ACG, Auto correlogram; AHP, Analytic Hierarchy Process; CF, Concentration field; CPC, Contribution pie chart; DHM, Distribution hotspot map; FCE, Fuzzy comprehensive evaluation; FDM, Finite Difference Method; GDM, Grade distribution map; GR, Geometric irregularity; IPC, Importance pie chart; LBM, Lattice Boltzmann Method; PBC, Periodic boundary conditions; RMH, Related matrix heatmap; TF, Temperature field; TSC, Time series chart; VF, Velocity field.

Synthesizing the research status outlined in Section 2.1, several interconnected challenges and obstacles become apparent. These challenges are not merely generic difficulties but are directly derived from the identified gaps, oversights, and methodological constraints prevalent in the current body of literature. Previous studies exhibit strong specificity but notable limitations (Figure 2). Research frameworks are often rigid and singular, neglecting the physical properties of indoor environments and the coupling of multiple physical fields. Additionally, most studies focus on wine, with limited attention to Chinese Baijiu, and oversimplify the complexities of boundary conditions in physical phenomena. These shortcomings stem from insufficient understanding of cellar environments' unique and shared characteristics, including their complexity, temporal variability, and coupled dynamics. In the Baijiu industry, empiricism dominates, with limited scientific rigor and universality in engineering practices, highlighting the need for innovative, science‐driven approaches. This study addresses these gaps by proposing thermal and humidity control techniques to accelerate aging processes. Given the overwhelming complexity of influencing factors and limited foundational data, it adopts a “Baijiu‐centered” approach. The goal is to comprehensively characterize cellar microenvironments under complex coupling effects, variable boundary conditions, and continuous dynamic changes. To systematically address the challenges mentioned above (Figure 2), we propose an integrated research framework, as shown in Figure 3.

Based on the systematic review of existing literature (Sections 2.1, 2.5) and the identification of key challenges (Section 2.2), we now shift from summarizing what is known to proposing what needs to be done. The following section presents a novel research framework designed to address the identified gaps. It is important to note that this framework is a prospective proposal—a set of testable hypotheses and methodological recommendations—rather than a synthesis of completed work. Its validity and feasibility remain to be demonstrated through future empirical research.

## 4 Future Directions

The novel framework aims to explore the potential of enhancing the aging process of Baijiu through environmental control measures, with a focus on the transport and coupling relationships between Baijiu and the external thermal‐humidity environment. It addresses the dynamic characterization, formation mechanism, and comprehensive evaluation of the microenvironment in storage cellars. The framework proposes a scientific method to accurately describe and assess the indoor microenvironment in unique underground air settings.

First, to monitor the dynamic changes in temperature and humidity, Apresys‐NFC tag‐type temperature and humidity recorders are used for continuous monitoring. Second, airflow velocity is continuously monitored using a hot‐wire anemometer. Then, non‐dispersive infrared (NDIR) sensors are employed to continuously monitor CO_2_ levels, reflecting the intensity of the fermentation activity while ensuring the safety of the air quality in the storage environment. Finally, gas chromatography–mass spectrometry (GC–MS) is used to accurately measure the volatile target gases in the base Baijiu, including the ‘atmosphere’ formed by volatile flavor compounds and impurities mentioned in the “volatile theory”. The project will also use data from the sensor network within the storage space for cross‐validation to improve data accuracy and reliability. Combined with temperature and humidity information, these measurement data allow for practical analysis of the “gas‐thermal‐humidity” environment and support targeted interventions to optimize Baijiu aging. By providing this operational detail, the framework not only remains at the conceptual model level but also demonstrates specific applicability to actual Baijiu aging systems.

### 4.1 The Novel Framework

Based on the above analysis, this paper highlights that a key future direction for Baijiu aging lies in emphasizing the physical properties of the environment to promote aging in a scientific manner. The primary task is to develop a comprehensive understanding of the cellar environment by integrating and coupling various physical factors and external influences. To address these challenges, this article proposes a novel framework aimed at advancing the scientific study and optimization of the Baijiu aging process.

Figure 3 involves investigating the functional identification, spatiotemporal distribution patterns, and dynamic transformation characteristics of target gases amid the uncertainties of base Baijiu volatilization and the randomness of flow diffusion. It examines the coupling relationships and mechanistic construction of the “gas‐thermal‐humidity” multi‐physical field under complex conditions, such as the irregular dimensions of ceramic vessels, their arrangements, and periodic soil boundary conditions. Furthermore, it aims to develop comprehensive evaluation methods for the aging room that account for multiple physical fields under continuous dynamic demands, including optimizing measurement points for improved accuracy.

Methodologically, this framework represents a significant advancement by deeply integrating multidisciplinary approaches. By combining engineering physics analysis with data‐driven and flavor‐oriented methods, it advances existing research and establishes a unified research paradigm, connecting building environmental science, multi‐physical fluid dynamics, spatiotemporal data analysis, and food flavor chemistry, systematically unifying these fields for use in the study of Baijiu aging.

Furthermore, the framework is designed to probe specific coupled mechanisms, such as how evaporation‐regulated humidity modulates gas‐phase reaction kinetics near the Baijiu surface or influences microbial ecology and activity within the porous jar mud seals. By integrating boundary‐specific measurements with multi‐physics modeling, the proposed approach moves beyond descriptive correlations towards a mechanistic understanding of the aging microenvironment.

#### 4.1.1 Dynamic Characteristics

**FIGURE 4:**
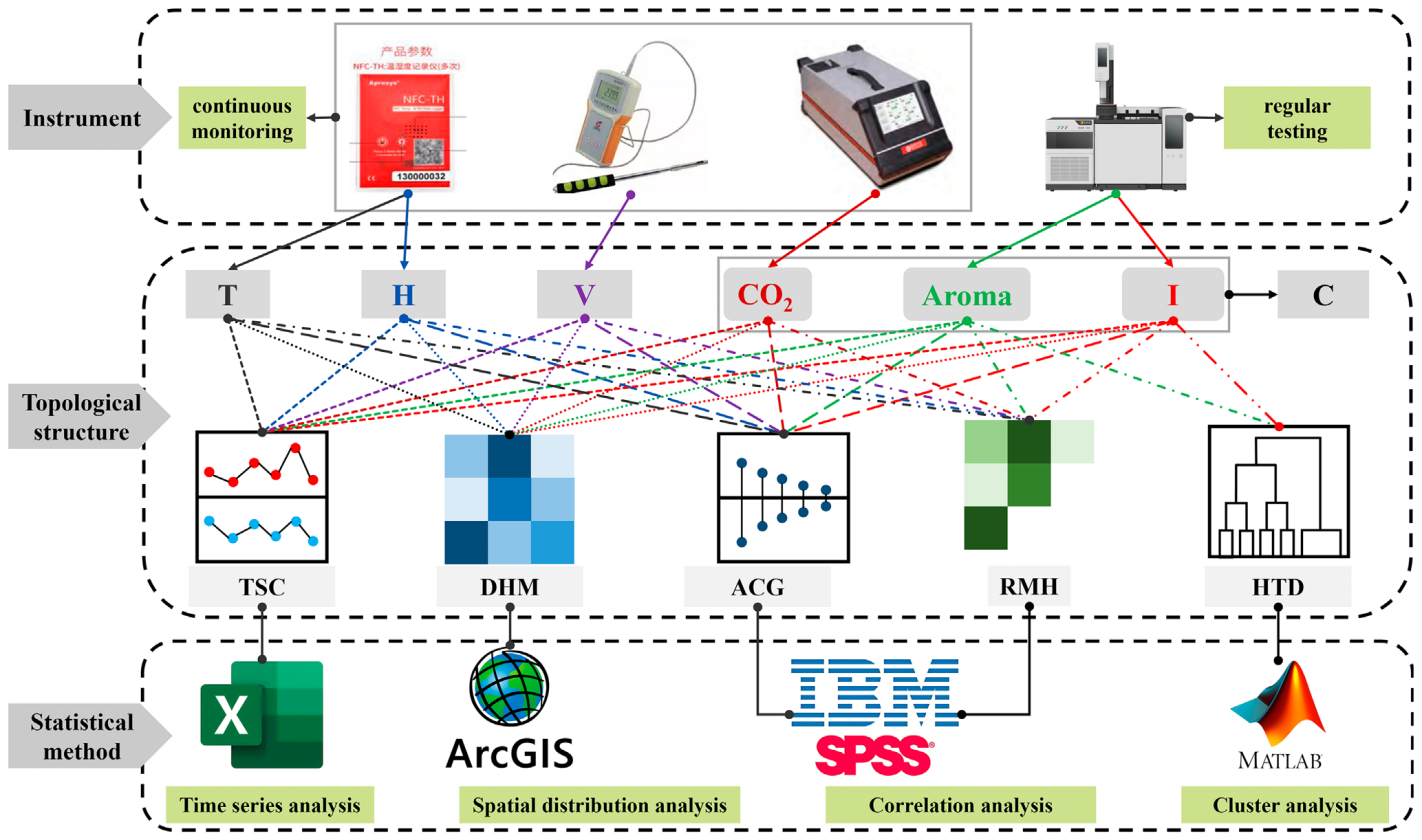
Test process and data analysis framework.

To address the challenges of insufficient understanding of spatiotemporal distribution patterns and incomprehensive environmental parameters identified in Section 2.2, amid the uncertainty of base wine volatilization, the novel framework focuses on the spatiotemporal distribution patterns of physical quantities such as temperature, humidity, and target gas concentrations. It analyzes the correlation of these physical quantities across both temporal and spatial dimensions to understand their dynamic transformation characteristics (Figure 4).

Initially, field measurements are conducted to collect data on temperature, humidity, airflow velocity, and CO_2_ concentrations, “aroma”, and impurity at various locations and times. Then, clustering analysis is utilized to classify and identify “aroma” and impurity gases. Time series analysis and spatial distribution analysis are employed to understand the dynamic changes in these data, leading to the creation of a comprehensive database.

Subsequently, statistical methods are applied to eliminate the effects of stochastic flow and deduce the coupling characteristics of multiple physical fields. The relationships between target gas concentrations and factors such as temperature and humidity, as well as the autocorrelation of all data, are examined. Ultimately, the correlation between the spatiotemporal distribution characteristics of the “Gas‐Thermal‐humidity” multi‐physical field and the flavor quality of base wine, alongside environmental factors, is uncovered.

#### 4.1.2 Regulation Mechanism

**FIGURE 5:**
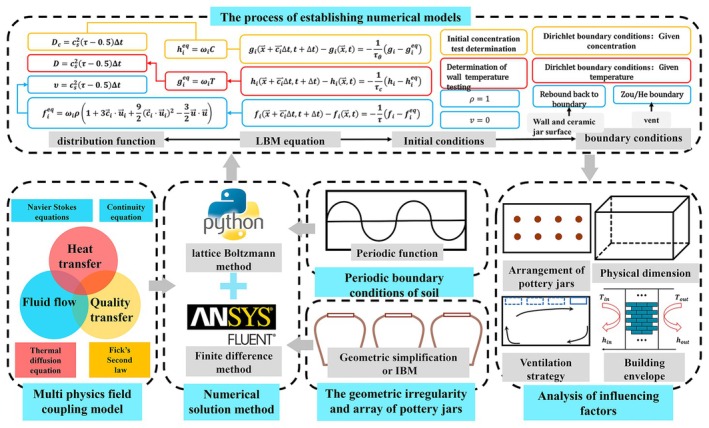
Numerical modeling framework for the “Gas‐Thermal‐Humidity” microenvironment: From governing equations to boundary conditions. Numerical modeling framework for the “Gas‐Thermal‐Humidity” microenvironment. The diagram illustrates: (a) the selection of numerical methods (Lattice Boltzmann Method, LBM) and their governing evolution equations; (b) the corresponding macroscopic conservation equations (Navier–Stokes, energy, and species transport); (c) the critical boundary conditions, with particular emphasis on the volatilization flux at the Baijiu surface and the porous media transport through the ceramic jar wall; and (d) the key coupling mechanisms between the thermal, flow, and concentration fields. The framework integrates periodic soil boundary conditions, jar arrangement geometries, and ventilation strategies to enable a comprehensive analysis of influencing factors.

In complex conditions, this framework aims to establish a “Gas‐Thermal‐Humidity” multi‐physical field coupling model to analyze the multiple physical fields influenced, thereby elucidating the mechanisms of “Gas‐Thermal‐Humidity” microenvironment (Figure 5). By constructing typical scenarios, the processes of mass transfer, heat transfer, and fluid flow will be repeatedly simulated to develop the multi‐physical fields coupling model. The framework systematically organizes and identifies known and unknown parameters in the model, including geometries, envelope properties, and environmental factors. Utilizing computational fluid dynamics, numerical simulation solutions for the proposed model will be developed to acquire fields such as temperature, velocity, and concentration. The model's accuracy will be validated through comparative analysis with measured data, and it will be refined based on experimental results to ensure reliability. The simulation investigates the influence mechanisms of factors such as temperature, humidity, ventilation, and the envelopes' properties on the multi‐physical fields.

#### 4.1.3 Evaluation System

**FIGURE 6:**
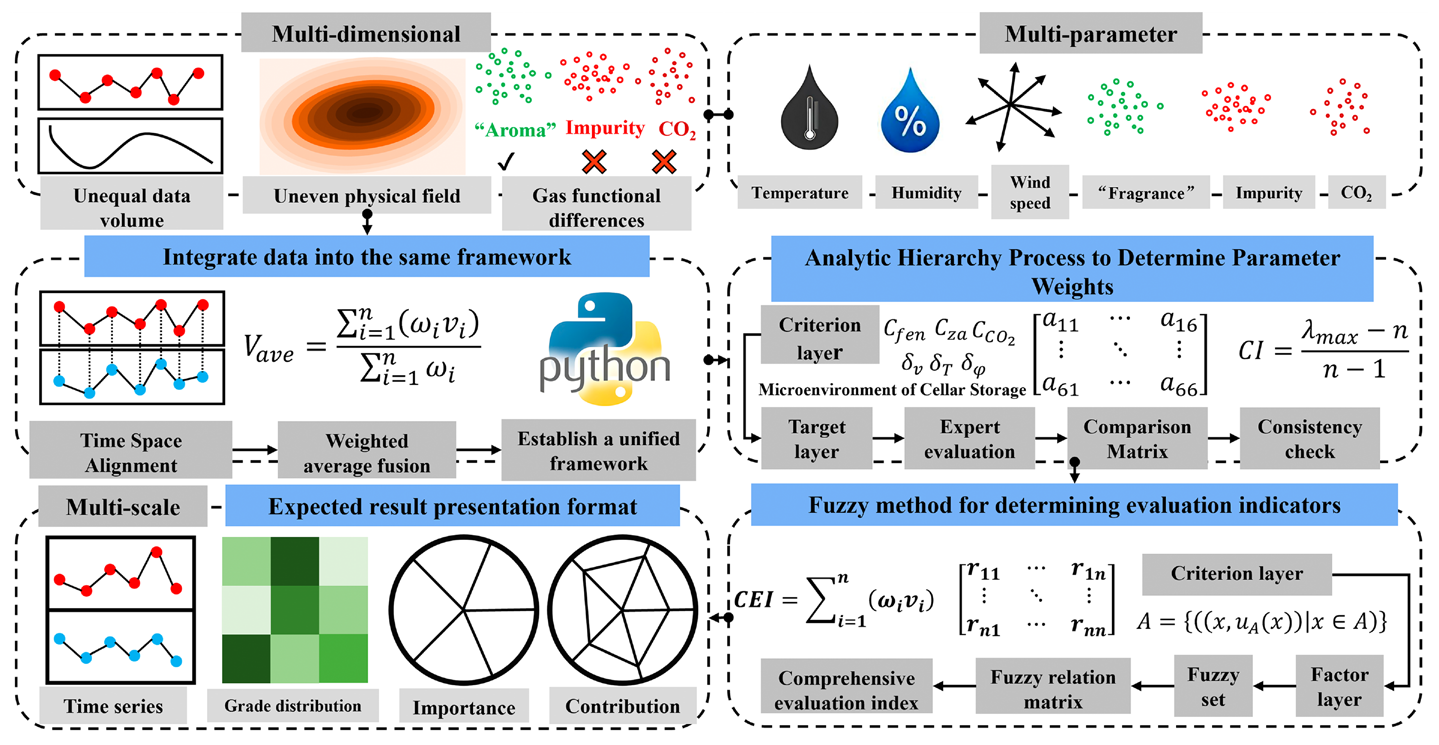
Multi‐parameter comprehensive evaluation framework for the cellar microenvironment based on AHP and fuzzy logic. Multi‐parameter comprehensive evaluation framework for the cellar microenvironment. The diagram illustrates: (a) the multi‐dimensional considerations underlying the evaluation; (b) the multi‐parameter monitoring indicators (temperature, humidity, wind speed, “fragrance”, impurity concentration, CO_2_); (c) the use of Analytic Hierarchy Process (AHP) to determine parameter weights at the criterion and factor layers; and (d) the application of fuzzy comprehensive evaluation (FCE) to integrate time‐series data, grade distributions, and parameter importance into a single Comprehensive Evaluation Index (CEI).

This framework addresses the need for continuous dynamic monitoring by focusing on two critical aspects: the heterogeneity of multi‐physical fields and the functional roles of target gases. It is designed to optimize measurement point layouts, improving monitoring efficiency and accuracy (Figure 6). The process begins with a comprehensive literature review to establish a theoretical foundation for evaluating aging microenvironments, summarizing commonly used environmental parameters and indicators. Based on this foundation, surveys, field measurements, and simulation analyses are conducted to identify critical parameters. These parameters are then assessed for importance, with corresponding weights calculated using appropriate methodologies. A comprehensive evaluation model for aging microenvironments is developed from these insights. Additionally, the framework creates databases capturing the spatial distribution and temporal trends of the evaluation index, forming a dynamic basis for ongoing analysis. Finally, by integrating geometries, ventilation dynamics, and temperature and humidity conditions, the framework systematically optimizes monitoring parameters, density, and layouts. This holistic approach enhances the accuracy and reliability of storage microenvironment evaluation and monitoring, ensuring robust insights for improving aging environments.

## 5 Conclusions

This study tackles the challenge of accelerating the aging process of Chinese Baijiu by focusing on the physical properties of the aging environment and the coupling interactions between gas exchange in the Baijiu and its surroundings. Using a systems science approach, it examines the existing knowledge framework, identifies critical challenges, and proposes a research framework rooted in the environmental characteristics. The key findings include:

(1) Environmental temperatures in underground cellars are not constant, with fluctuations in amplitude and unevenness that warrant further exploration.

(2) Current research focuses predominantly on volatile gases within the Baijiu, with minimal attention to gases released into the surrounding air.

(3) The study introduces the “gas‐heat‐humidity” microenvironment concept, offering a systematic research framework and implementation pathway to guide future work.

The study acknowledges its limitations: (1) dependence on insights from wine aging research due to limited studies on Baijiu aging environments, (2) an unverified hypothesis regarding gas distribution that requires experimental validation, and (3) the proposed framework is primarily aimed at the design of underground or semi‐underground ceramic jar aging systems; its applicability to other storage methods, such as stainless steel tanks in warehouses, may require adjustments and further study.

## Author Contributions

**Jin Li:** conceptualization, methodology, writing – original draft. **Yuwen Wen:** systematic review, formal analysis. **Chang Yi:** data collection, software, analysis. **Wenwu Zhou:** data collection, validation. **Yin Zhang:** validation, visualization, project administration, writing – review and editing.

## Funding

This work is financed by the Chinese Scholarship Council (No. 202410890003), the Key Research Base Program of the Sichuan Social Sciences Association of China (No. 25SCZSXW02), and the College Students' Innovative Entrepreneurial Training Plan Program, Sichuan University of Science and Engineering (No. cx2025162).

## Disclosure

Declaration of Generative AI in Scientific Writing: The authors declare that they did not use any AI or AI‐assisted technologies in the manuscript preparation.

Submission Declaration and Verification: The authors declare that this work has not been published previously, that it is not under consideration for publication elsewhere, that its publication is approved by all authors and tacitly or explicitly by the responsible authorities.

## Conflicts of Interest

The authors declare no conflicts of interest.

## Data Availability

Data will be available on request.
